# A Technical Approach to the Evaluation of Radiofrequency Radiation Emissions from Mobile Telephony Base Stations

**DOI:** 10.3390/ijerph14030244

**Published:** 2017-03-01

**Authors:** Raimondas Buckus, Birute Strukčinskienė, Juozas Raistenskis, Rimantas Stukas, Aurelija Šidlauskienė, Rimantė Čerkauskienė, Dorina Nicolina Isopescu, Jan Stabryla, Igor Cretescu

**Affiliations:** 1 Faculty of Medicine, Vilnius University, M. K. Čiurlionio Str. 21, LT-03101 Vilnius, Lithuania; juozas.raistenskis@vuvl.lt (J.R.); rimantas.stukas@mf.vu.lt (R.S.); sidlauskiene.aurelija@gmail.com (A.Š.); rimante.cerkauskiene@gmail.com (R.Č.); 2 Faculty of Health Sciences, Klaipeda University, Manto Str. 84, LT-92294 Klaipeda, Lithuania; birutedoctor@hotmail.com; 3 Faculty of Civil Engineering, “Gheorghe Asachi” Technical University of Iaşi, 65, Mangeron Blvd, 700050 Iaşi, Romania; dorina_isopescu@yahoo.co.uk; 4 Faculty of Technical Sciences, University of Warmia and Mazury in Olsztyn, 10-719 Olsztyn, Poland; 5 Faculty of Chemical Engineering and Environmental Protection, “Gheorghe Asachi” Technical University of Iaşi, 73, Mangeron Blvd., 700050 Iaşi, Romania

**Keywords:** electric field strength, power density, non-ionizing electromagnetic radiation, mobile telephony base station antenna

## Abstract

During the last two decades, the number of macrocell mobile telephony base station antennas emitting radiofrequency (RF) electromagnetic radiation (EMR) in residential areas has increased significantly, and therefore much more attention is being paid to RF EMR and its effects on human health. Scientific field measurements of public exposure to RF EMR (specifically to radio frequency radiation) from macrocell mobile telephony base station antennas and RF electromagnetic field (EMF) intensity parameters in the environment are discussed in this article. The research methodology is applied according to the requirements of safety norms and Lithuanian Standards in English (LST EN). The article presents and analyses RF EMFs generated by mobile telephony base station antennas in areas accessible to the general public. Measurements of the RF electric field strength and RF EMF power density were conducted in the near- and far-fields of the mobile telephony base station antenna. Broadband and frequency-selective measurements were performed outside (on the roof and on the ground) and in a residential area. The tests performed on the roof in front of the mobile telephony base station antennas in the near-field revealed the presence of a dynamic energy interaction within the antenna electric field, which changes rapidly with distance. The RF EMF power density values on the ground at distances of 50, 100, 200, 300, 400, and 500 m from the base station are very low and are scattered within intervals of 0.002 to 0.05 μW/cm^2^. The results were compared with international exposure guidelines (ICNIRP).

## 1. Introduction

Measurements and evaluation of radiofrequency (RF) electromagnetic radiation (EMR) levels, the RF EMR exposure guidelines recommended for the general public, and the establishment of other regulations are issues of worldwide importance [1,2]. At the moment, the most important concern is the contradictory information about mobile telephony base station antennas and their declared effects on human health [3,4].

After reviewing the scientific literature, in 1998 the International Commission for Non-Ionizing Radiation Protection (ICNIRP) formulated guidelines on exposure guidelines for RF EMFs in the frequency range from 0 Hz to 300 GHz. These guidelines are based on acute health effects such as elevation of tissue temperatures resulting from absorption of energy during exposure to RF EMFs between 100 kHz and 300 GHz [5]. The results of studies on possible long-term effects such as cancer were not considered to be adequate for setting limits on a scientific basis.

In 1999, the European Union published recommendations on the exposure guidelines of the general public to RF EMFs. These recommendations rely on the international exposure guidelines (ICNIRP) of 1998 and are therefore based on scientific appraisal of risk-related data. Some countries have established similar national laws, regulations, guidelines, or standards for exposure to radio frequency fields, while others have adopted or are in the process of adopting the ICNIRP guidelines in Europe in response to the EU recommendations. Accordingly, most national and international documents are based on the concept of avoiding the established short-term health effects of exposure [5,6,7].

The European Commission recommendation adopted on 12 July 1999 requires that a maximum field strength of RF EMR (0–300 GHz) be established and that information about the population’s exposure to RF electromagnetic fields (EMFs) and the measures taken to reduce them be provided [7]. RF EMF intensity parameters are measured for a variety of reasons:

(a) Radio engineering installations are measured in order to evaluate RF EMFs produced by them. Such measurements are done in accordance with safety hygiene norms, standards, or other regulatory documents;
(b) RF EMF measurements are carried out according to requests by the general public, authorities, or suppliers. The measurements are conducted in a particular place (for example, in a room, balcony, playground, etc.) by identifying RF EMF sources;
(c) Comparative measurements of RF EMFs, produced by a radio engineering facility in one location, are compared against those in another one;
(d) Scientific RF EMF studies are based on long-term monitoring measurements covering measurement locations that are most frequented by people, and the obtained data are used for epidemiological studies [8,9].

The precise experimental determination of the power density of antennas in complex environments is a difficult task. This is mainly due to the existence of three fundamental physical properties of electromagnetic waves: reflection, absorption, and interference [10,11,12]. Under uncontrolled conditions, for instance in a complicated environment, different measurements can lead to quite different results due to changing conditions [13,14,15]. Moreover, the settings of the measurement equipment may affect the accuracy of the measured values. Special attention should be paid to the exposure assessment methodology [16].

Reducing the highest allowed level of RF EMR, and the establishment of other legislation on High Frequency Electromagnetic Dosimetry, are issues of worldwide importance, taking into consideration the contradictory information about mobile phones, mobile telephony base stations, and their possible dangerous effects on human health [17,18,19,20,21].

In performing field measurements of RF EMFs, the key task is to appropriately identify measurement locations [22]. An important stage is to evaluate where the values of RF EMF intensity parameters will be the highest [23]. This can be done using orthophotos, various maps, or modelling programs, or by measuring the areas concerned using an RF EMF meter and identifying and selecting zones with the highest RF EMF values [24].

In order to identify RF EMF propagation limits and adjustment areas in a specific locality, it is necessary to appropriately select the main parameters of antenna spatial distribution: the height of the antenna’s geometric centre, the direction of the main radiation (main lobe), and the required downtilting of the directional pattern on the vertical and horizontal planes [25,26]. However, evaluation of the main components of the physical environment (relief, buildings, etc.) restricting RF EMR propagation is problematic. Furthermore, it is difficult to estimate RF EMF values on building facades and to calculate the total propagation values of several antennas [27,28].

RF EMR measurements are carried out according to standards, norms, references, and legal rights specific for radiation generated by mobile telephony base station antennas [29]. However, it is difficult to evaluate the specific features of RF EMR according to the description. Accordingly, in order to evaluate which antenna provides a radiation level spread in the environment, detailed RF EMR investigations need to be carried out and measurement points need to be multiplexed [30,31,32].

However, field measurements carried out according to the requirements do not always reflect the real dispersion of mobile telephony base station antennas’ RF EMR in the environment [33]. When conducting field measurements on RF EMR, it is very important to foresee and evaluate the areas in which field measurements will be conducted. One of the most important parts is to evaluate where the values of RF EMR will be highest, that is, to determine the direction of the main radiation (main lobe) from each antenna [34,35,36]. This is done in two steps: by analysing the technical characteristics of mobile telephony base station antennas and by conducting field measurements. In order to evaluate the direction of the main radiation, all of the directional diagrams of the antenna in the vertical and horizontal planes are used. All mobile telephony antenna manufacturers provide antenna radiation diagrams with technical characteristics presented in specialised catalogues [36,37,38,39].

Selecting a model that will enable understanding of the principal qualities, structure, development tendencies, and interaction with the environment of the RF EMF is of particular importance. A properly created pattern not only helps with managing the RF EMFs of the antenna, but also assists in predicting direct and indirect consequences of practical applications (e.g., impacts on other objects or the environment) [40]. Simulation is required for both existing and intended objects and makes it possible to predict the conditions under which RF EMR meets the required value but still does not exceed the permitted limits [41].

RF EMR produced by antennas for mobile telephony can be experimentally measured with the help of different software applications employed to simulate the reference values of RF EMR [42]. The simulation of the RF EMR of mobile telephony includes the analysis of one or several different antennas emitting RF EMR, which would be difficult or impossible to research in normal conditions, using their emission models [43,44,45]. The simulation must evaluate parameters such as the radiation diagram (direction) of the antenna for mobile telephony, amplification, polarization, power, frequency, landscape openness, land relief, the height of buildings, the distance between buildings, the angle of the direction of the dispersed signal, the impact of signal diffraction on rooftops and reflections, the operating wavelength, and other factors [46,47,48,49,50]. In order to analyse the power density of the RF EMF under changing terrain, a number of different environmental factors must be assessed [51].

The simulation of the RF EMR of mobile telephony is based on the finite-difference time-domain method, according to which quite a few common software packages aimed at analysing, simulating, and designing RF EMR have been created. The available software applications include Fidelity, REMS, XFDTD, SEMCAD, EmGine Environment, Concerto, “CST“ MWS, Cellular Expert, MSC Cell Tool V2, and Satimo [48]. The main objective of this article is to evaluate the dispersion of RF EMR in the environment generated by mobile telephony base station antennas.

## 2. Materials and Methods

Characterization of the parameters of RF EMR from mobile communications in the near-field of the antenna covers measurements of electric field strengths. The RF EMF power density is measured in the far-field. We evaluated the measurement points by measuring the areas concerned with an EMF meter and we present the zones with the highest RF EMF values. The influence of the other base stations in the vicinity is very small (about 1%), so this was not taken into account.

Evaluation of the electric fields measurements was done according to LST EN 50383:2010 and LST EN 50492:2009. Only at a sufficiently large distance from the source, in the so-called far-field region, is it sufficient to evaluate the RF EMF power density in microwatts per centimeter squared [50,51]. The parameters of the investigated mobile telephony base station antennas are presented in Table 1.

The chosen mobile telephony base station antennas are limited to bands around 900 MHz (GSM), 1800 MHz (further GSM services) as well as 1900–2200 MHz (UMTS). (GSM = Global System for Mobile Telecommunication, UMTS = Universal Mobile Telecommunications System).

### 2.1. Measurements in Near-Field and Far-Field Areas

A theoretical calculation was carried out: the field distribution can be calculated using the parameter Lz = 2D^2^/λ, where D is the dimensions of the antenna’s active (radiating) part, λ is the wavelength, and Lz denotes the transitional field range. According to the calculation, we chose a sufficiently large distance (30 m) from the source. So, in the near-field area, that is closer than 30 m from the antenna (in both the horizontal and the vertical plane), the strengths of the electric fields are measured. In the far-field areas, that is further than 30 m (in both the horizontal and the vertical plane), the RF EMF power density is measured from the antenna. If the mobile telephony base station antenna is mounted on a building or mast that is higher than 30 m, the RF EMF power density is measured on the ground. The influence of the other base stations in the vicinity is very small (about 1%), so they are not defined. For multiple frequency sources the exposure quotient (*EQ_tot_*) was used to evaluate the public exposure:

$$EQ_{tot}={\sum_{i=1}^{N}\left(\frac{E_{i}^{meas}}{E_{i}^{guide}}\right)}^{2}$$

where *N* = total number of radio frequencies in the evaluation point; *E_i_^meas^* = value of electrical field component, related to the corresponding frequency *i*; *E_i_^guide^* = limit value of electrical field component, according to the guide related to the corresponding frequency *i*; If *EQ_tot_* is less than 1, then the environment is considered safe against radio frequency radiation [52].

All measurements were performed between 9:00 and 16:00 via a field meter placed on a tripod. Measurements at each selected point were performed in triplicate. Measurements were performed at different heights from the floor, namely 1, 1.5, and 1.7 m. Measurements were done in the far-field of the macrocell mobile telephony base station antennasby using a NBM 550 broadband field meter (Narda, Hauppauge, NY, USA) with an EF 0392 isotropic E-probe and a Narda SRM 3006 selective radiation meter with a 27 MHz–3 GHz isotropic E-probe.

The results presented in Figures 7, 8 and 13 were obtained using a professional broadband field meter (Narda NBM 550) measuring the electric field strength (V/m) (the instantaneous field strength of all signals presented in a large frequency band). The broadband field meter cannot distinguish between signals coming from different transmitting sites. However, this does not cause a great problem when the measuring field levels are in the frame of the exposure guidelines recommended by ICNIRP regulations.

The results presented in Figures 9–12 and 14 were obtained with a selective radiation meter (Narda SRM 3006).

“Spatial Averaging” was used for exposure measurements. Spatial averaging is the technique used to measure field exposure (E) and power density (S) averaged over a number of spatial locations. The results recorded at each measurement point include average values over 6 min and the highest field strength value was recorded as the measurement value of the selected point.

Uncertainties of the probes and devices were determined from the calibration certificates corresponding to the measurement devices (Table 2).

### 2.2. Description of Antennas and Locations

Field measurements of the RF EMF power density and electrical field strength of a mobile telephony base station antenna were carried out at Šeškinės st. 2, where the KATHREIN 80010292 antenna is located.

Field measurements of electrical field strength were carried out in the near-field, that is, up to 30 m distance (in both the horizontal and the vertical plane) from the antenna. Field measurements of the RF EMF power density were carried out at more than 30 m distance from the antenna. The mobile telephony base station antenna stands 14 m above the earth’s surface, having the following coordinates: 54°42′45.78″ N and 25°15′0.56″ E (WGS coordinate system).

**Figure 1:**
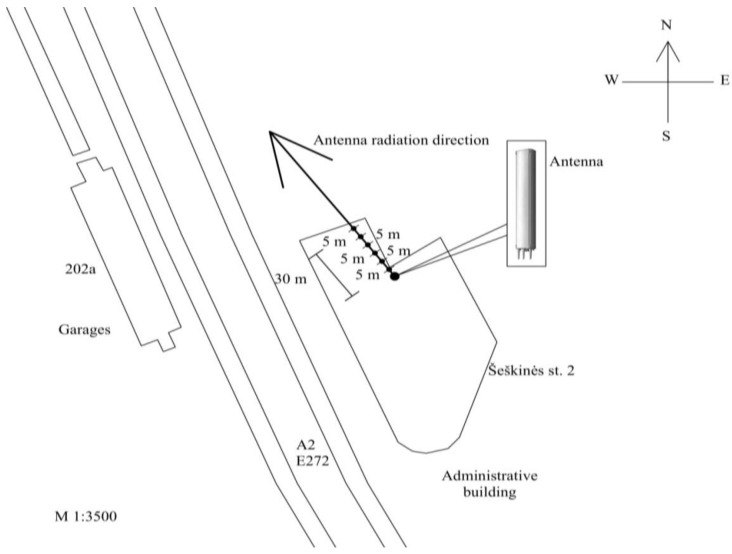
Scheme of the electric field strength of the mobile telephony base station antenna (1739 W ERP) on the roof.

First, measurements of the electrical field strength were carried out in the near-field (up to 30 m distance) on the roof in the direction of the main radiation (main lobe) from the mobile telephony base station antenna according to Figure 1. RF EMF intensity parameters were measured at different distances of 1, 5, 10, 15, 20, 25, and 30 m.

**Figure 2:**
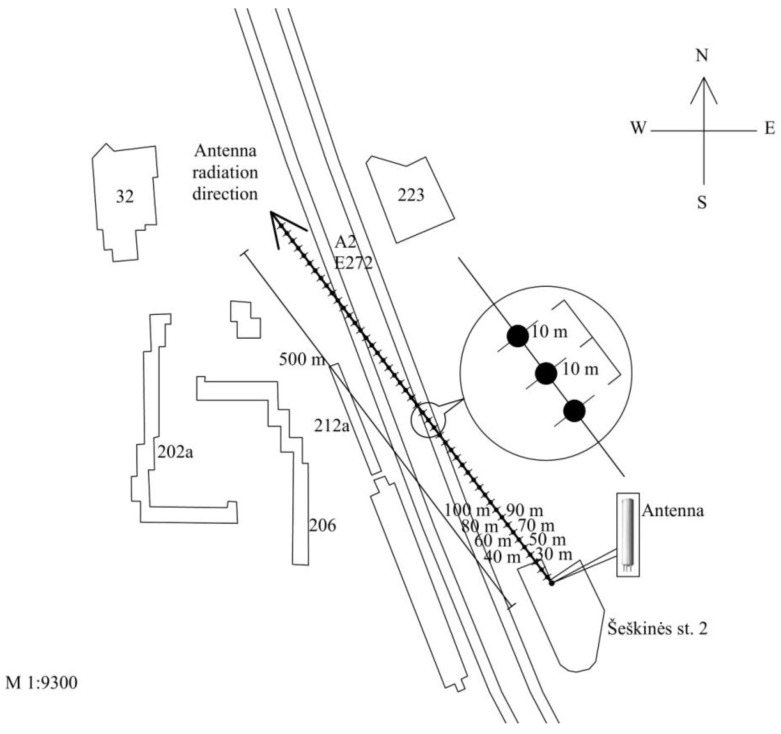
Measurement scheme of electromagnetic power density of the mobile telephony base station antenna (ERP = 1739 W).

The antenna points north-west. The azimuth of maximum radiation is 290°. The antenna works at three frequencies (900/1800/2100): in the frequency ranges of 950–954 MHz (GSM-900 technology), 1847–1850 MHz (GSM-1800 technology), and 2137–2138 MHz (UMTS-2100 technology). The maximum effective radiated power is 1739 W, the coverage zone in the horizontal plane is 65°/65°/65°, the coverage zone in the vertical plane is 7.5°/7.6°/6.8°, the mechanical downtilt is 0/°0°/0°, the electrical downtilt is −5°/−5°/−5°, and the gain is 17.5/17.5/17 decibels isotropic (dBi). Secondly, according to the scheme in Figure 2, measurements of the RF EMF power density were carried out in the far-field area (more than 30 m distance) on the ground in the direction of the main radiation (main lobe) from the KATHREIN 80010292 mobile telephony base station antenna (the parameters of the antenna are the same). The RF EMR measurement points were located at intervals of 10 m: the first measurement point was on the ground 30 m from the antenna’s vertical axis, the second at 40 m, and so on up to 500 m.

Measurement locations were selected in the road, field, or car park. The azimuth of all measurement points was 290°.

**Figure 3:**
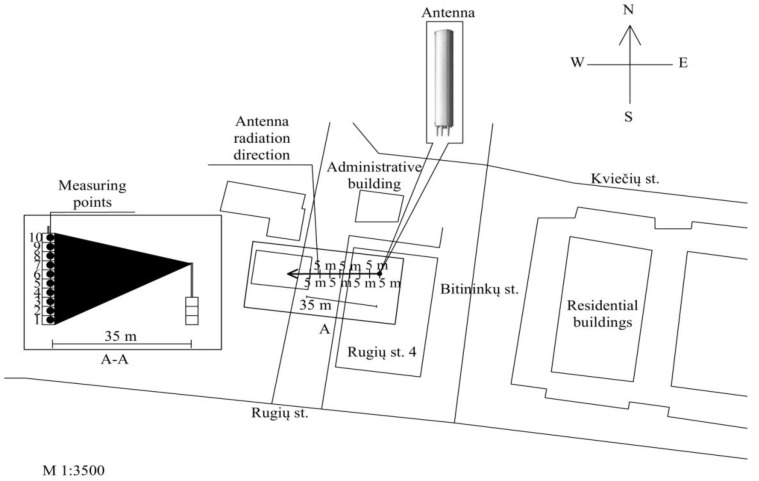
Measurement scheme of electromagnetic power density of the mobile telephony base station antenna (ERP = 1428 W).

According to the scheme shown in Figure 3, the field measurements of the RF EMF power density were carried out on every floor of the 10-storey building. The direction of the main radiation (main lobe) from the KATHREIN 742241 antenna was towards the 10-storey building. Measurements of the RF EMF strength were not carried out due to the distance between the measurement points and antenna, which was more than 30 m. The distance between the building and the antenna was 35 m. The antenna worked at three frequencies (900/1800/2100): in the frequency ranges of 951–953 MHz (GSM-900 technology), 1836–1840 MHz (GSM-1800 technology), and 2137–2138 MHz (UMTS-2100 technology). The effective radiated power was 1428 W, the coverage zone in the horizontal plane was 65°/65°/65°, the coverage zone in the vertical plane was 7.5°/7.8°/7.3°, the mechanical downtilt was 0/°0°/0°, the electrical downtilt was −4°/−4°/−4°, and the gain was 17/16.8/17 dBi. The antenna was mounted on a 10-storey building. The antenna’s pole height was 9.5 m and its length was 2 m. The azimuth of all measurement points was 250° and the corresponding coordinates were 54°44′31.15″ N and 25°16′15.56″ E.

**Figure 4:**
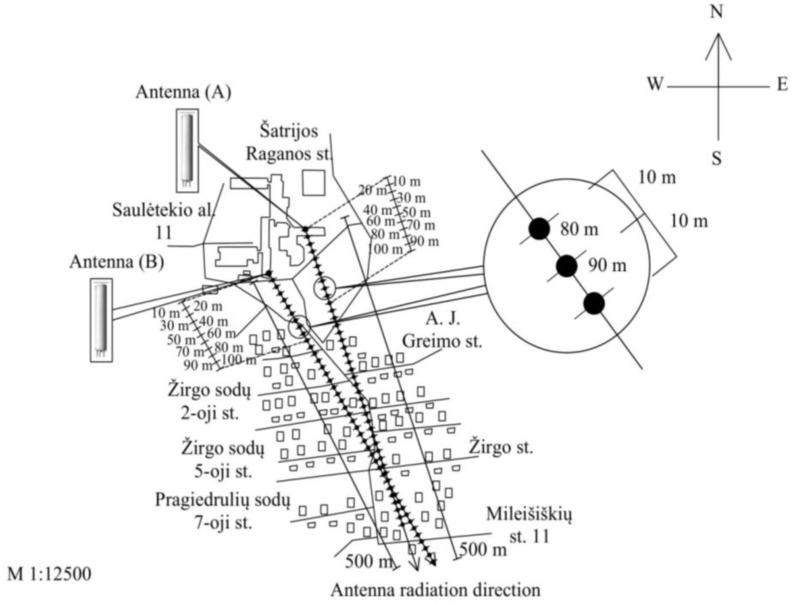
Measurement scheme of the electromagnetic power density of the mobile telephony base station antenna (ERP = 1329 W).

According to the scheme shown in Figure 4, measurements of the RF EMF power density were carried out on the ground in the direction of the main radiation (main lobe) from the KATHREIN 742241 antenna. The antenna works at three frequencies: in the frequency ranges of 950–954 MHz (GSM-900 technology), 1836–1847 MHz (GSM-1800 technology), and 2137–2138 MHz (UMTS-2100 technology). The effective radiated power was 1328 W, the coverage zone in the horizontal plane was 65°/65°/65°, the coverage zone in the vertical plane was 7.5°/7.8°/7.3°, the mechanical downtilt was 0/°0°/0°, the electrical downtilt was −1°/−2°/−2°, and the gain was 17/168/17 dBi. The antenna was located on a 30 m high public building. The length of the antenna was 2 m. RF EMR measurement points were located at intervals of 10 m: the first measurement point was on the ground at a distance of 10 m from the antenna’s vertical axis, the second at 20 m, and so on up to 500 m. The locations of measurements were selected in the road, field, or car park. The azimuth of the measurements was 150°.

RF EMF power density measurements were carried out on the ground in the direction of the main radiation (main lobe) from the KATHREIN 742266 antenna. The antenna works at two frequencies: in the frequency ranges of 944–958 MHz (GSM-900 technology) and 2122–2123 MHz (UMTS-2100 technology). The total effective radiated power was 707 W, the coverage zone in the horizontal plane was 65°/68°, the coverage zone in the vertical plane was 7.5°/5°, the mechanical downtilt was 0/°0°, the electrical downtilt was −4°/−4°, and the gain was 16.5/17.8 dBi. The antenna was located on a 30 m high building. The antenna’s pole height was 2 m and its length was 2.6 m. RF EMR measurement points were located at intervals of 10 m: the first measurement point was on the ground at a distance of 10 m from the antenna’s vertical axis, the second at 20 m, and so on up to 500 m. The azimuth of measurements was 170°.

**Figure 5:**
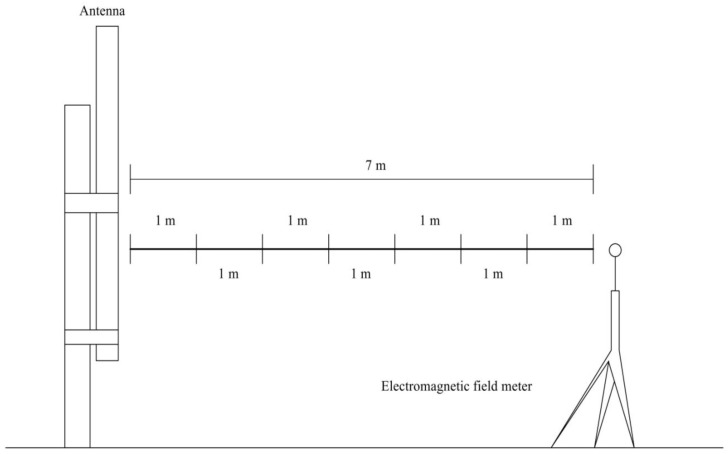
RF EMF power density measurement scheme for the mobile telephony base station antenna (ERP = 1329 W) (the measurement points correspond to different distances from the antenna’s vertical axis, located in the centre of the roof).

Measurements of the electric field strength were also carried out in the near-field (up to a distance of 7 m) on the roof (the measurement location’s height was 30 m and its address was Sauletekio st. 11) in the direction of the main radiation (main lobe) from the KATHREIN 742241 antenna according to Figure 5.

**Figure 6:**
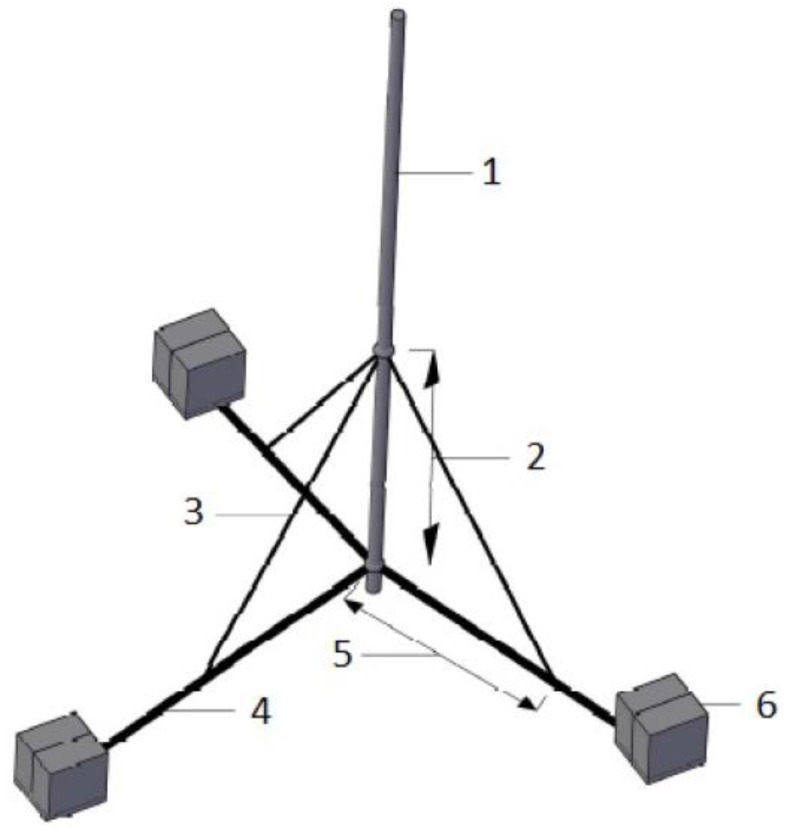
The bearing support for the mast: 1. mast; 2. height of fastening system; 3. brace; 4. bearing support branch; 5. length of fastening system; 6. concrete blocks.

A detailed possible anchorage (supporting the antenna on the roof) designed to withstand a characteristic wind speed of, for example, 100 km/h is presented. The antenna is fixed upright on the mast, and, by means of the fastening system, has a limited possibility of automatic adjustment through a crank angle between 0° to 10°. To fix the mast (with a diameter of less than 70 mm), the bearing support shown in Figure 6 can be used.

## 3. Results of Case Study Research

**Figure 7:**
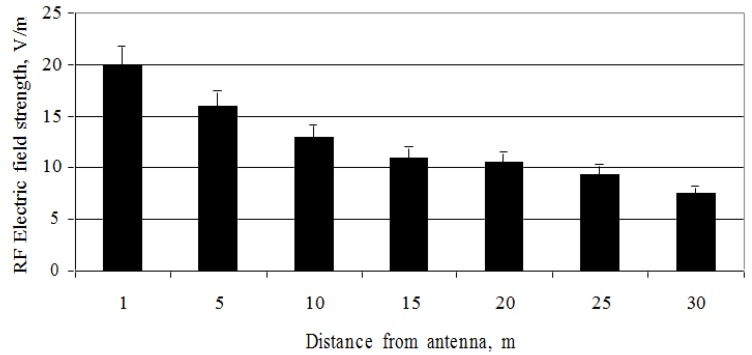
Distribution of the RF electric field strength of mobile telephony base station antenna (ERP = 1739 W) at different distances.

Field measurements of the directional mobile antenna (ERP = 1739 W) at a height of 12 m above the ground showed that the electric field strength rapidly declined with increasing distance from the antenna (Figure 7).

An intense decrement of the strength of the electric field is noticeable at 15 m distance from the mobile antenna, where its values are decreased almost by half, from 20 to 11.4 V/m. At 20 m or more from the antenna, the strength of the electric field is not so intense and fluctuates between 10.5 and 7.5 V/m. The maximum electric field strength was measured as 20 V/m and was half of the ICNIRP exposure guidelines.

The investigation of the directional mobile antenna (ERP = 1329 W, at 31 m height above the ground) revealed that a more intense and stronger fluctuation of the electric field strength occurred at the height of the mobile antenna’s geometric centre: the values of the electric field strength at distances between 1 and 7 m were distributed in the interval ranging between 161 and 35 V/m.

**Figure 8:**
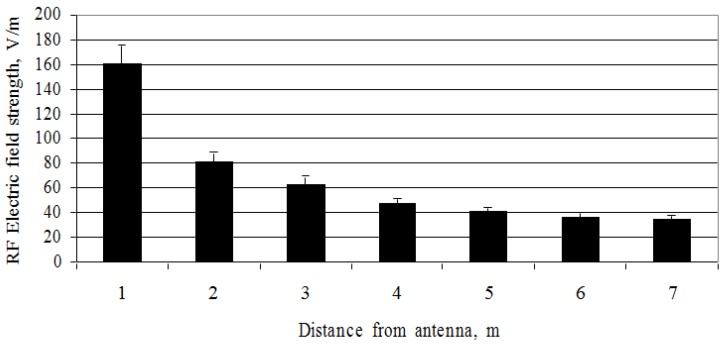
Distribution of the RF electric field strength at different distances from the mobile telephony base station antenna (ERP = 1329 W).

On comparing the values of the electric field strength of the directional mobile antenna (geometric centre height = 14 m, ERP = 1739 W) at a height of 12 m above the ground with those of the directional mobile antenna (geometric centre height = 31 m, ERP = 1329 W) at a height of 31 m above the ground, it is noticed that the intensity of the RF EMF parameters varied by a factor of approximately 10 (Figure 7 and Figure 8). The field measurements conducted clearly show that the RF EMF intensity parameters depend directly on the vertical distance from the height of the antenna’s geometric centre. The maximum electric field strength was measured as 160 V/m, which was 2.6 times higher than the reference level.

**Figure 9:**
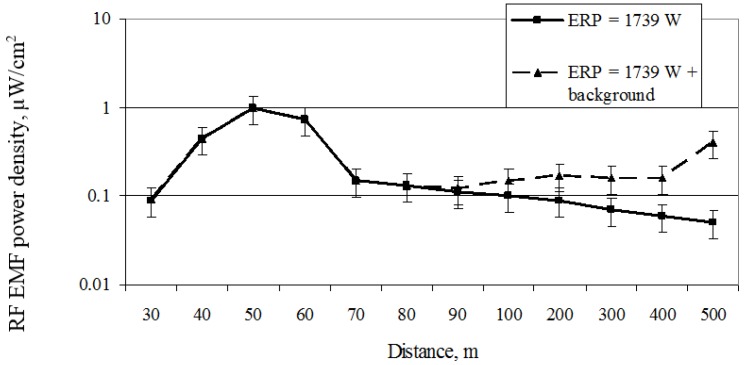
Distribution of the RF power density at different distances from the mobile telephony base station antenna (ERP = 1739 W) and other radio engineering objects (background).

Figure 9 shows that the maximum local RF EMF power density (0.98 µW/cm^2^) created by the directional mobile antenna (ERP = 1739 W, height = 12.7 m) in the direction of the main radiation (main lobe) occurs at a height of 1.5 m above the ground and a distance of 50 m. At distances greater than 50 m, the RF EMF power density begins to decrease: at 60 m distance it reaches 0.74 µW/cm^2^, at 70 m it is 0.15 µW/cm^2^, at 80 m it is 0.13 µW/cm^2^, at 90 it is 0.11 µW/cm^2^, and at 100 m it is 0.1 µW/cm^2^. At a distance of more than 100 m, the electromagnetic power density fluctuates very marginally and is distributed in the interval between 0.05 and 0.1 µW/cm^2^. The field measurements showed that the antenna diagram in the vertical plane has a relatively narrow radiation-width radiation slope of 6.8°, which is directed downwards because of the mechanical and electrical downtilt. Accordingly, the greatest power density appears where the main antenna’s diagram slip reaches the earth‘s surface (in this case at a distance of 50 m from the antenna). Obviously, the created exposure limit of the mobile antenna depends on the antenna’s ERP and its height above the earth’s surface.

In order to determine the influence on the total RF EMF power density values of each mobile antenna technology and the general RF EMR background (radio, TV broadcasting, and other telecommunication antennas are included under “background”), investigations were carried out at particular mobile antenna frequencies. The background RF EMR value was found by subtracting the RF EMR from the mobile antenna at the emitted frequencies from the general RF EMR level investigated.

**Figure 10:**
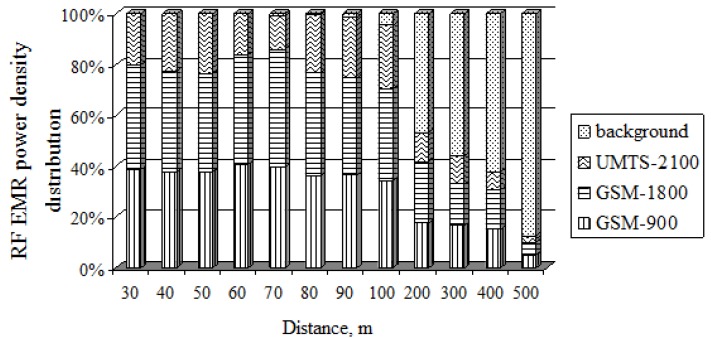
Contributions of different antenna types to the total exposure.

Figure 10 illustrates the contributions to the RF EMR distribution of the GSM-900, GSM-1800, and UMTS-2100 mobile antennas (ERP = 1739 W, height = 14 m) and the background depending on distance. From the figure it is clear that the RF EMR at different frequencies emitted by the directional mobile antenna dominated up to a distance of 90 m. The power density of the background RF EMF contributed approximately 0.01% of the total RF EMR. The biggest part of the RF EMR (up to 72%) consisted of the RF EMF power density created in the radio frequency bands of the GSM-900 MHz and GSM-1800 MHz mobile antenna technologies. The RF EMF power density created in the UMTS-2100 MHz radio frequency bands amounts to about 28% of the general RF EMR. The uneven distribution of the RF EMR of the three technologies depends on each technology’s RF EMR effective power: The GSM-900 MHz radio frequency band’s ERP is 698 W, the GSM-1800 MHz radio frequency band’s ERP is 736 W, and the UMTS-2100 MHz radio frequency band’s ERP is 305 W. From the field measurements conducted, it is easy to see that the background RF EMR has an increasing influence at distances of 100 m or more from the mobile antenna: at 100 m distance, the background RF EMR amounts to 7% of the total RF EMR, at 200 m it is 50%, at 300 m it is 58%, at 400 m it is 65%, and at 500 m it is 92%. Background RF EMR consists of television, radio, and telecommunications base station antennas in the vicinity of the investigated area.

**Figure 11:**
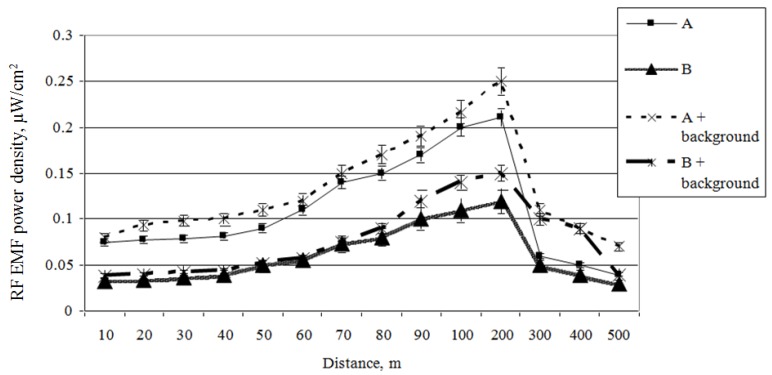
Contributions of different antenna types to the total exposure.

**Figure 12:**
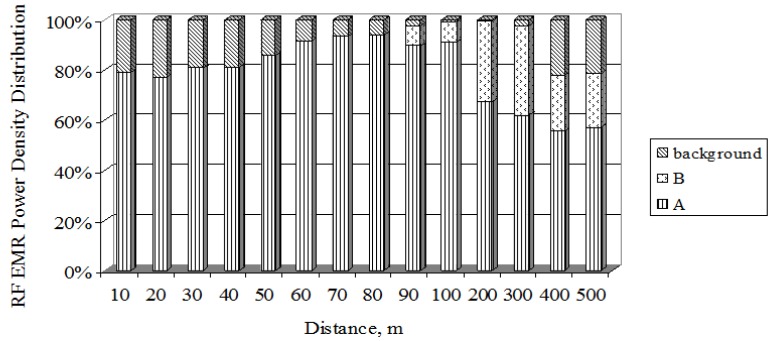
Contributions of different antenna types to the total exposure.

In order to evaluate the influence of two mobile antennas on the total RF EMR in a particular area, measurements of the RF EMF power density of directional antennas A (ERP = 1329 W, height = 30 m) and B (ERP = 707 W, height = 30 m) were carried out on mobile antennas at a height of 1.5 m above the earth’s surface (Figure 11 and Figure 12).

The calculated EQ is less than 1 and therefore the environment is considered safe against radio frequency radiation.

The results plotted in Figure 11 show that the maximum local RF EMF power density created by the mobile antennas are in a 200 m distance, but these exposure guidelines are not as strong as their numerical value (because of the lower ERP and greater height). This influences the form of the mobile antenna’s directional diagram in the vertical plane, with the main power density reaching the earth’s surface at a distance of 200 m. At distances of 200 m or more from the antenna, the RF EMF power density declines in inverse proportion to the distance from the antenna’s geometric centre to the investigated point and fluctuates between 0.25 and 0.03 µW/cm^2^. At greater distances, where the greatest interference by electromagnetic waves, absorption, and reflection occurs, the power density of the RF EMF declines even more intensely. Further away from the mobile antenna’s pole axis RF EMF, the power density is distributed gradually at a height of 1.5 m above the earth’s surface (this is approximately the height at which mobile telephony are usually used).

It is evident that the RF EMR created by mobile antenna A (ERP = 1329 W) is twice as intense as that created by mobile antenna B (ERP = 707 W). It is difficult to evaluate each antenna’s influence on the total RF EMR according to a given figure, and therefore each antenna’s RF EMF power density distribution layer is presented (Figure 12).

Figure 12 shows that in the direction of the main radiation (main lobe) from mobile antenna A (ERP = 1329 W), the RF EMR created by the antenna itself dominates. In the antenna’s zone (up to a distance of 50 m), increased background RF EMR clearly dominates comparing to antenna A. This can be explained by the fact that opposite to mobile antenna A (ERP = 1329 W), at a distance of 60 m from it, stands another mobile antenna, whose radiated RF EMF crosses that of the investigated antenna. At distances of 90 m and more, the influence of the RF EMF power density created by mobile antenna B (ERP = 707 W) on the total field increases. The largest influence of mobile antenna B (ERP = 707 W) (about 40%) on the total field occurs at a distance of 200–300 m from the antenna. Because of the different azimuths of the antennas, when moving further away, the influence of mobile antenna B (ERP = 707 W) on antenna A (ERP = 1329 W) decreases, whereas the background RF EMR increases.

### Investigations in the Vertical Plane

**Figure 13:**
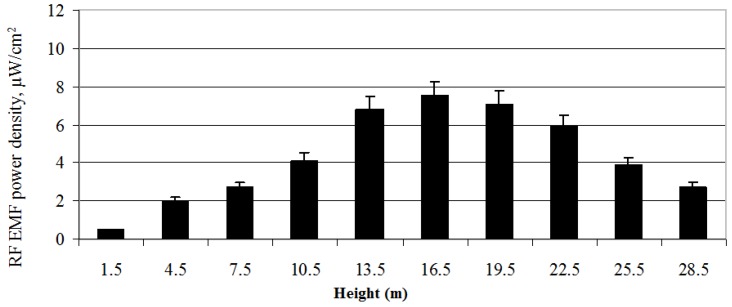
Distribution of the electromagnetic power density of the mobile telephony base station antenna (ERP = 1428 W) at different heights.

The distribution of the RF EMF power density of the mobile antenna (ERP = 1428 W, height of 21.5 m) in the vertical plane is illustrated in Figure 13.

It is evident that the mobile antenna (ERP = 1428 W, height = 21.5 m) at a distance of 35 m and a height of 13 to 23 m creates a 20-m basic radiation (coverage) zone in the vertical plane. At this height, the values of the power density of the RF EMF are distributed in the interval between 5.5 and 7.5 µW/cm^2^. As shown, the maximum value of the RF EMF power density occurs at a height of 16.5 m, but is not at the same level as the antenna’s geometric centre, that is, at a height of 21.5 m above the ground. It is determined by the basic antenna characteristics of the directional diagram in the vertical plane. This diagram is a parameter that defines at what height and how widely the antenna radiates RF EMR. The distribution of the mobile antenna’s diagram depends on the antenna’s electrical and mechanical downtilt angle [23]. The mobile antenna’s (ERP = 1428 W, height = 21.5 m) directional diagram in the vertical plane is formed by inclining the antenna at the angle of the electrical downtilt (–4°). The antenna’s downward direction forms the basic coverage zone between the heights of 13 and 23 m. The electromagnetic power density limits were not exceeded.

**Figure 14:**
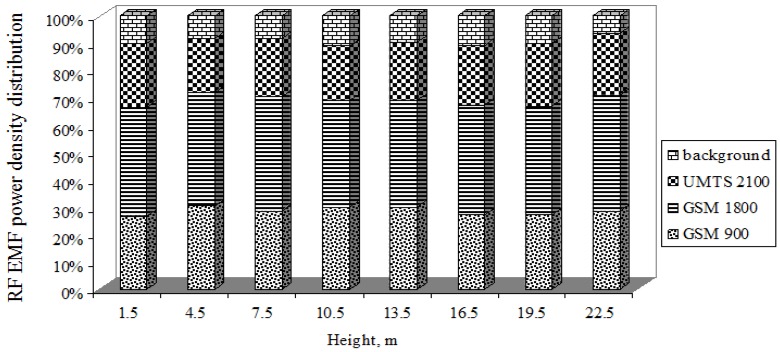
Contributions of different antenna types to the total exposure.

Figure 14 illustrates the contributions of the GSM-900, GSM-1800, and UMTS-2100 mobile antenna technologies (ERP = 1428 W, height = 21.5 m) and background to the RF EMR distribution, as a percentage, depending on height. As the figure shows, the distributions of the GSM-900 MHz, GSM-1800 MHz, and UMTS-2100 MHz mobile antenna technologies’ RF EMR and background RF EMR are practically the same for all possible cases of height. If we compare the percentage RF EMF power density distribution from distance with the percentage RF EMF power density distribution from height, we can see that when moving further away from the mobile antenna, the background RF EMR increases (Figure 14), whereas when the height increases above the ground, the background radiation changes very little. This proves that the RF EMF power density created by the mobile antenna, depending on the electric and mechanical downtilt angles, is distributed in the horizontal plane.

The uneven RF EMR distribution of the mobile antenna (ERP = 1428 W, height = 21.5 m) depends on the antenna technology used. This mobile antenna uses three technologies, each characterized by different RF EMR as follows: for the GSM-900 technology, the ERP is 407 W; for the GSM-1800 technology it is 675 W; and for the UMTS-2100 technology it is 346 W. The calculated EQ is less than 1 and therefore the environment is considered safe against radio frequency radiation.

## 4. Discussion

Measurements of RF electromagnetic field intensity parameters can be used to determine compliance with exposure guidelines. The great number of base stations are mostly installed in populated areas. Base stations operating in densely populated cities in the GSM 900, 1800 and UMTS 2100 MHz frequency bands are continuously evolving due to the conditions of competition and the need for a better response to increasing customer demand. This evolution is carried out by replacing classic antennas with new high-gain antennas or establishing a new base station in the targeted area. Naturally, the electromagnetic field density of the environment has also changed in parallel with this evolution process. Due to these changes, people who are working near base stations, especially on the roof, could be exposed to an electromagnetic field strength that is several times higher than for people working farther away from the base stations. Excessive exposure of people to electromagnetic fields can be avoided by correct positioning of the base station antennas.

Rowley et al. [16] studied the distribution of the values of the electric field of a 35 m high mobile antenna at a height of 29–35 m (ERP = 1500 W) in the near zone (up to 30 m distance) and found that the values of the electric field strength were distributed between 0.15 and 42.47 V/m. The distribution of the values of the electric field strength depends on the distribution of the antenna diagram in the vertical plane. On comparing the results of the field measurements conducted with other results, it is obvious that the largest convergence of the results is obtained when the antenna height reaches 31 m, at which the values of the electric field strength at the distance of 1–30 m are distributed in the interval between 5 and 20 V/m.

Field-measurement scientists carried out a study on mobile telephony bases station antennas located on roofs [13]. Their measurements established that the strength of the electric field emitted by a mobile antenna at a distance of 1 m reached 77.9 V/m; that emitted by a mobile antenna with ERP = 160 W at a distance of 1 m reached 26.5 V/m, and that emitted by a mobile antenna with ERP = 1350 W at a distance of 1 m reached 128 V/m. The values obtained for the electric field strength in our study are similar to these. The main difference is the mobile antenna’s ERP, which is dependent on the electric field strength in the near zone.

Some recent studies clearly illustrate the directional mobile antenna’s feature of creating a local RF EMF power density at a certain distance, with values in the frame of the maximum exposure guidelines [37]. Mobile antennas 20 m high (with ERP = 800 and 1000 W, respectively) create an RF EMF power density exposure limit proportionately to distances of 60 and 85 m, respectively. In the first case, the power density value was equal to 1.8 µW/cm^2^, and in the second it was equal to 1 µW/cm^2^.

The discrepancy in the RF EMF power density values during the field measurements conducted can be explained by the fact that antennas with different levels of effective radiation were chosen for investigation. The distance at which the exposure limit is reached depends on the electric and mechanical downtilts.

Alhekail have studied RF EMR from mobile antennas. With his colleagues, he established that each antenna (using the GSM-900, GSM-1800 and UMTS-2100 technologies) creates unequal RF EMR values at particular frequencies [42]. His group established that 20%, 40%, and 40% of the total RF EMR is contributed by mobile antennas (ERP—800 W, height = 30 m) in the 900, 1800, and 2100 MHz bands, respectively. As can be seen from the comparison, the frequency ranges of different antennas, according to the percentage contribution to the total RF EMR found by a field measurement survey, differ from the results of this study. This is influenced by the environment that surrounds the mobile antenna: the 900 MHz frequency band is used more often in rural areas and rarely in populated areas, whereas the 1800 and 2100 MHz frequency bands are used more often in densely populated cities. Alhekail investigated mobile antennas [42] in the densely populated city of Riyadh in Saudi Arabia (where the distance between the antenna and residential buildings was 50 m, while in the present study the distance between the antenna and residential buildings was 200 m). Usually, in the monitoring of field measurements, each antenna generating RF EMR is investigated separately, but the total RF EMR created by two mobile antennas has not been evaluated. Furthermore, no investigations of each antenna’s contribution to the total RF EMR have been carried out.

The methodology to conduct the field measurements, described in the presented study case research can be further used for evaluation of the epidemiological state of a certain area such as: metropolitan area, urban agglomeration area, etc.

## 5. Conclusions

In this research study case performed in Vilnius (the largest urban community in Lithuania) an original investigation methodology for RF EMF assessment was proposed, taking into consideration all presented technical aspects, which could be of interest to specialists in mobile telephony base station antennas and public health, respectively. The measurements of the RF electric field strength and RF EMF power density conducted in the near- and far-fields of the mobile telephony base station antennas, lead to the following conclusions:

(1) In the near-field (up to a distance of 30 m), the values of the strength of the RF electric field radiated by the antennas varied from 7 to 195 V/m. In the near-field, the RF EMR intensity parameters exceeded the allowable norm (ICNIRP Guidelines);
(2) The RF EMF power density values of the high effective radiation power emitted into the environment by the mobile antenna up to a distance of 500 m amount to approximately 0.01%–10% of the allowable level (10 µW/cm^2^). This is influenced by the effective radiation power of the mobile antenna, the downtilt angle, and the height of the antenna above the earth’s surface;
(3) The high effective radiation power of the directional mobile antenna revealed that the antenna with a height of 30 m has small values of RF EMF power density, reaching about 0.01%–0.04% of the allowable level (10 µW/cm^2^);
(4) The RF EMF power density values of the mobile antenna with a height of 14 m at a distance of 50 m reached 0.98 μW/cm^2^, but decreased significantly at greater distances (100 m or more), and amounted to only 0.005%–0.01% compared with the ICNIRP allowable values;
(5) The RF EMF power density values on the ground surface at a distance of 50–200 m from the mobile antenna were very small and varied from 0.01 to 0.98 μW/cm^2^, which is 100 to 10 times lower than the allowable level (10 µW/cm^2^);
(6) The RF EMF power density values of mobile antennas with high effective radiation power and a height of up to 30 m are significantly higher and reach 1 μW/cm^2^. This influences the direction diagrams of mobile antennas in the vertical plane. In this position, where the basic diagram’s slip reaches the earth’s surface, topical maxima are formed. Depending on the antenna’s height and electrical and mechanical downtilt, the maxima of the power density of the RF EMF can form at a distance of 100–300 m;
(7) The RF EMF power density values of mobile telephony base station antennas in the air at ground level increase farther from the antenna. The highest values can be observed at the point where the main antenna radiation zone reaches the earth’s surface; farther away, these decline.

## Figures and Tables

**Table 1:** Antenna technical specifications.

Effective Radiated Power (ERP), W	Height, m	Type	Technology	Measurement Location (Accessible to the General Public)
1739	12.7 *	KATHREIN 80010292	GSM-900, GSM-1800, UMTS 2100	On the roof area and on the ground
1428	20.5 **	KATHREIN 742241	GSM-900, GSM-1800, UMTS-2100	In residential area
1329	33.3 ***	KATHREIN 742241	GSM-900, GSM-1800, UMTS-2100	On the roof area and on the ground

* Total: 12.7 m (from the ground to the top of the antenna) = building: 9 m + antenna mast: 2.4 m + the middle of the antenna panel: 1.3 m; ** Total: 20.5 m (from the ground to the top of the antenna) = building: 10 m + antenna mast: 9.5 m + the middle of the antenna panel: 1 m; *** Total: 33.3 m (from the ground to the top of the antenna) = building: 30 m + antenna mast: 2 m + the middle of the antenna panel: 1.3 m.

**Table 2:** Uncertainties of probes and devices.

**EF 0392 Isotropic E-Probe**		**SRM 3006 E Probe**	
**Frequency (MHz)**	**Uncertainty (dB)**	**Frequency (MHz)**	**Uncertainty (dB)**
0.1–300	0.5	26–400	1
300–1000	1	400–1500	1
1000–3000	0.5	1500–3000	1
**Manufacturer Data Sheet**			
Flatness of frequency response (Calibration uncertainty not included)	1	Flatness of frequency response (Calibration uncertainty not included)	1
Linearity	1.5	Linearity	2
Isotropic response	1	Isotropic response	1
Temperature response	0.5	Temperature response	0.5
